# Gold Nanoparticles Inhibit VEGF_165_-Induced Migration and Tube Formation of Endothelial Cells via the Akt Pathway

**DOI:** 10.1155/2014/418624

**Published:** 2014-06-01

**Authors:** Yunlong Pan, Qing Wu, Li Qin, Jiye Cai, Bin Du

**Affiliations:** ^1^Department of General Surgery, The First Affiliated Hospital of Jinan University, Guangzhou 510632, China; ^2^Department of Histology and Embryology, Medical School of Jinan University, Guangzhou 510632, China; ^3^Department of Chemistry, Life Science and Technology School of Jinan University, Guangzhou 510632, China; ^4^Department of Pathology, Medical School of Jinan University, Guangzhou 510632, China; ^5^Division of Clinic Pathology, The First Affiliated Hospital of Jinan University, Guangzhou 510632, China

## Abstract

The early stages of angiogenesis can be divided into three steps: endothelial cell proliferation, migration, and tube formation. Vascular endothelial growth factor (VEGF) is considered the most important proangiogenic factor; in particular, VEGF_165_ plays a critical role in angiogenesis. Here, we evaluated whether gold nanoparticles (AuNPs) could inhibit the VEGF_165_-induced human umbilical vein endothelial cell (HUVEC) migration and tube formation. AuNPs and VEGF_165_ were coincubated overnight at 4°C, after which the effects on cell migration and tube formation were assessed. Cell migration was assessed using a modified wound-healing assay and a transwell chamber assay; tube formation was assessed using a capillary-like tube formation assay and a chick chorioallantoic membrane (CAM) assay. We additionally detected the cell surface morphology and ultrastructure using atomic force microscopy (AFM). Furthermore, Akt phosphorylation downstream of VEGFR-2/PI3K in HUVECs was determined in a Western blot analysis. Our study demonstrated that AuNPs significantly inhibited VEGF_165_-induced HUVEC migration and tube formation by affecting the cell surface ultrastructure, cytoskeleton and might have inhibited angiogenesis via the Akt pathway.

## 1. Introduction

It is a well-known fact that angiogenesis plays important role in cancer and other diseases and vascular endothelial growth factor (VEGF) is a crucial regulatory molecule during angiogenesis [1, 2]. The early stages of angiogenesis can be divided into three steps: endothelial cell proliferation, migration, and tube formation [3]. As an isoform of VEGF, VEGF-A regulates the key steps of the angiogenic process, particularly endothelial cell proliferation and migration [4, 5]. This cytokine promotes endothelial cell migration by interacting with its receptor VEGFR-2 and activating the downstream VEGFR-2/PI3K/Akt/eNOS axis in endothelial cells [5, 6]. VEGF-A has two main isoforms: the heparin-binding growth factor VEGF_165_ and the non-heparin-binding growth factor VEGF_121_; these isoforms are the most important proangiogenic factors during angiogenesis [4].

In recent years, several anti-VEGF strategies have been explored in clinical settings. The most clinically advanced inhibitors were the recombinant humanized monoclonal antibodies against VEGF, including bevacizumab [7]; however, the clinical applications of these antibodies are greatly limited because of the high cost and incidence of side effects [8]. Currently, research regarding the use of nanomaterials to inhibit angiogenesis is emerging rapidly [9]. Given their small particle size, good biocompatibility, and low toxicity, gold nanoparticles are considered a good prospect for biological applications [10, 11].

Several reporters have demonstrated that gold nanoparticles (AuNPs) exhibited potentially antiangiogenic effects by interacting with the heparin-binding domain of VEGF_165_ [12]. Our previous study showed that AuNPs inhibited VEGF_165_-induced HUVEC proliferation and contributed to reduced levels of tumor angiogenesis [13]. However, the effects of AuNPs on endothelial migration and tube formation remained unknown.

The present study was conducted to demonstrate the effects of AuNPs on endothelial migration and tube formation. In this study, we found that AuNPs suppressed VEGF_165_-induced endothelial cell migration and tube formation and might have inhibited these processes through the Akt signaling pathway. Furthermore, we showed that AuNPs altered the cell membrane surface ultrastructure, a phenomenon that might be associated with angiogenesis. The results suggested that AuNPs reduced angiogenesis by repressing endothelial cell migration and tube formation.

## 2. Materials and Methods

### 2.1. Preparation and Characterization of AuNPs

In a typical experiment [14], 0.01 mol/L of chloroauric acid (5 mL) and 1% sodium citrate (10 mL) were added to 50 mL of an aqueous solution that had been heated and stirred until reaching the boiling point; heating and vigorous stirring were maintained until the solution reached a wine red color. The newly formed AuNPs were filtered through a 0.22 *μ*m filter, used in the experiments, and ultimately characterized via UV-Vis absorption spectroscopy and transmission electron microscopy.

### 2.2. HUVEC Isolation and Culture

The HUVECs were harvested from human umbilical veins via 0.25% trypsin (Gibco, NY, USA) digestion. After washing in D-Hanks solution, the umbilical veins were perfused with a 0.25% trypsin solution for 10 min at 37°C, after which the solution was collected and centrifuged for 10 min at 1000 rpm/min. The cells were passaged via trypsin digestion and cultured in M199 media (Gibco, USA) containing 10% fetal bovine serum (Gibco, USA), 2 mM glutamine (Gibco, USA), and 1% penicillin/streptomycin (Sigma, USA) in an incubator at 37°C and 5% CO_2_.

The HUVECs were treated with VEGF_165_ or AuNP-treated VEGF_165_. AuNPs (125 or 250 nM) were prepared in sterile distilled water, diluted to the required concentrations in cell culture medium, and incubated with VEGF_165_ (20 ng/mL) overnight at 4°C before the experiments. Cells in the control group were cultured in basal medium.

### 2.3. Wound-Healing Assay

An in vitro wound-healing assay was performed to measure unidirectional HUVEC migration [15]. The HUVECs were seeded in 6-well plates (1 × 10^5^ cells/well), incubated in M199 with 10% FBS for 24 h, and subsequently washed twice with PBS and incubated in M199 with 1% FBS. After the cells grew to confluence, a straight line was scratched across the culture with a 10–200 *μ*L micropipettor tip. The cells were treated with VEGF_165_ (20 ng/mL) in the presence or absence of AuNPs (125 or 250 nM) and were incubated at 37°C and 5% CO_2_. Images of the cells were obtained with an inverted phase contrast microscope (Olympus, Tokyo, Japan) at 0 h and 24 h. The wound width was determined with ImageJ software.

### 2.4. Transwell Monolayer Permeability Assay

HUVEC migration was evaluated with a Transwell system (Corning Costar, MA, USA) that comprised 8 *μ*m polycarbonate filter inserts in 24-well plates [16]. Briefly, serum-starved cells were trypsin-harvested in M199 with 0.5% FBS. Next, 600 *μ*L of medium containing 1% FBS and VEGF_165_ (20 ng/mL) with/without AuNPs was added to the lower chambers, while HUVECs (1 × 10^5^) were plated in the upper chambers. After 12 h incubation, the cells on the bottom of the Transwell membrane were fixed with 4% paraformaldehyde at 37°C for 20 min and stained with 1% crystal violet at 37°C for 10 min; the nonmigrating cells in the upper chamber were removed with blunt-end swabs. The membranes were washed three times with PBS and photographed under a fluorescence microscope (Olympus; Tokyo, Japan). Finally, the crystal violet was dissolved in 33% acetic acid, and the absorbance was measured at 600 nm. The amount of cell migration was determined as the ratio of the OD values of the treatment relative to the control. Each treatment was repeated in 4 independent chambers.

### 2.5. Capillary-Like Tube Formation Assay

The formation of HUVECs into capillary-like structures on Matrigel (BD Biosciences, Bedford, MA, USA) was evaluated as previously described [9, 16]. HUVECs were pretreated with VEGF_165_ (20 ng/mL) in the presence or absence of AuNPs (125 or 250 nM) in starvation medium at 37°C in a humidified atmosphere of 5% CO_2_ for 24 h. At least 30 min before the experiment, 96-well plates were coated with Matrigel. Next, trypsin-harvested HUVECs were seeded onto the plated Matrigel (1 × 10^4^ cells per well) in serum-free M199 medium and incubated at 37°C. Images of the formation of capillary-like structures were obtained after 6 and 12 h with a computer-assisted microscope (Olympus, Tokyo, Japan) at 100x magnification. Tubular structures were quantified by manually counting the numbers of connected cells in randomly selected fields at 100x magnification; the total tube number in the control group was designated as 100%.

### 2.6. Chick Chorioallantoic Membrane (CAM) Assay

The effects of the test compounds on angiogenesis were investigated with the CAM assay as described previously [9]. Briefly, groups of 10 fertilized chicken eggs were incubated at 37°C and 80% humidity. On the sixth day of incubation, a square window was opened in each shell. Filter paper disks saturated with VEGF_165_, AuNP-treated VEGF_165_, or PBS were placed on the areas between preexisting vessels, after which the embryos were incubated for an additional 48 h. After the second incubation, the CAM arterious branches in each treatment group were photographed and counted using a Nikon digital camera system (Chiyoda-ku, Tokyo, Japan). The antiangiogenic effect of the AuNPs was indicated by relative numbers of arterious branches. The assay was performed three times to ensure reproducibility.

### 2.7. Single-Cell AFM Measurement

HUVECs were seeded onto slides and treated with VEGF_165_ (20 ng/mL) in the presence or absence of AuNPs (250 nM) for 24 h, after which the cells were fixed with 4% paraformaldehyde for 15 min, washed three times with PBS, and air-dried at room temperature. An AFM (Autoprobe CP Research, Veeco, USA) was used in the contact mode to obtain topographic images in air [17]. The silicon nitride tips (UL20B; Park Scientific Instruments) used in all AFM measurements had been ultraviolet-irradiated in air for 15 min to remove any organic contaminants prior to use. The tip curvature radius was <10 nm, and the cantilever length, width, and thickness were 115, 30, and 3.5 mm, respectively, with an oscillation frequency of 255 kHz and a force constant of 0.01 N/m (manufacturer's information). The prepared sample was placed on the AFM XY-scanning station, and more than 10 cells were measured. The acquired images were processed only using the software provided with the equipment (Image Processing Software Version 2.1, IP 2.1) to eliminate low-frequency background noise in the scanning direction (flattened order: 0-1).

### 2.8. Western Blot Analysis

Akt and phospho-Akt antibodies were obtained from Cell Signaling Technology (Beverly, MA, USA). The GAPDH antibody was obtained from Kangchen (Shanghai, China). Goat anti-mouse IgG and anti-rabbit IgG were obtained from Calbiotech (San Diego, CA, USA). HUVECs were plated into a 6-well plate (3 × 10^5^ cells/well). After adhering, the cells were starved and incubated in serum-free medium for 24 h and then treated with VEGF_165_ (20 ng/mL) in the presence or absence of AuNPs (125–250 nM) for another 24 h. Whole-cell lysates were collected and boiled for 10 min in 2x SDS sample buffer, subjected to 10% SDS-PAGE, and transferred to PVDF membranes (Amersham Life Sciences). The blots were blocked in blocking buffer (5% nonfat dry milk/1% Tween-20 in TBS) for 1 h at room temperature and then incubated with the primary antibodies (anti-Akt, 1 : 5000 dilution; anti-phospho-Akt, 1 : 500; and anti-GAPDH, 1 : 10,000) in blocking buffer for 2 h at room temperature. The bands were then visualized using horseradish peroxidase-conjugated secondary antibodies (1 : 2000) and ECL (Pierce Biotech, Rockford, IL, USA). The experiments were repeated three times.

### 2.9. Statistical Analysis

All data were presented as the means ± SEM from at least three independent experiments. Statistical analyses were performed with SPSS 17.0 software. Comparisons between the groups were performed with the two independent samples' *t*-test; those between multiple groups were tested with one-way ANOVA, and those between any groups were tested with SNK. A probability value of *P* < 0.05 was considered statistically significant.

## 3. Results

### 3.1. Characterization of AuNPs

**Figure 1:**
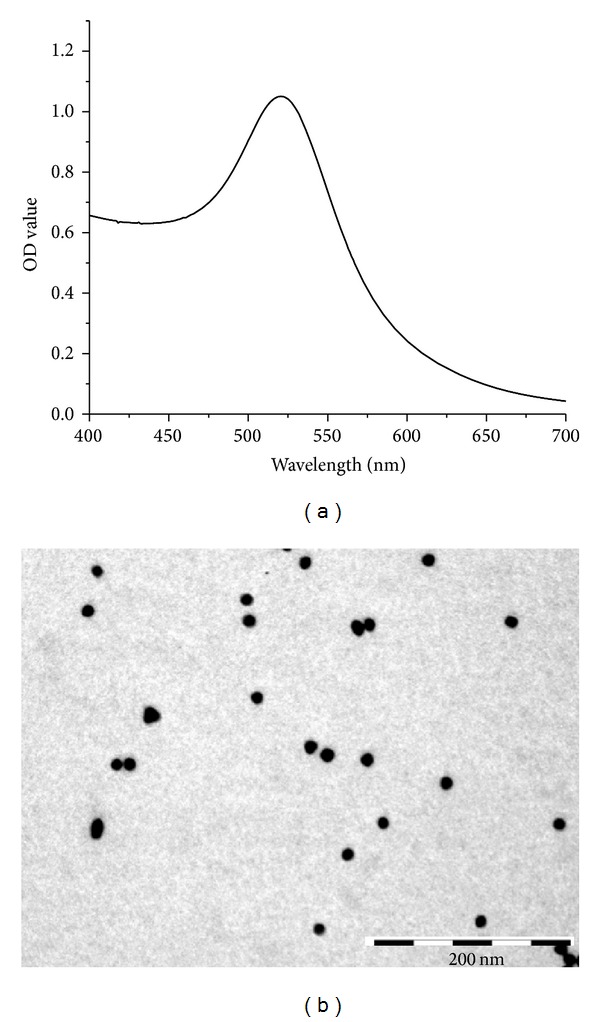
UV-Vis spectrum and TEM image of the AuNPs. (a) Purified AuNPs were analyzed using UV-Vis absorption spectroscopy. (b) The sizes of the AuNPs in a 15 nm diameter range.

The AuNPs were characterized using UV-Vis spectrophotometry and TEM. The AuNPs were red wine-colored and exhibited a peak in the 520 nm region of the UV-Vis spectrum (Figure 1(a)). Next, TEM was used to define the size distribution of the AuNPs. The monodisperse AuNPs were found to be spherical with a mean diameter of 15 nm (Figure 1(b)). Purified AuNPs were used for further studies.

### 3.2. Effects of the AuNPs on VEGF_165_-Induced HUVEC Migration

It is a well-known fact that endothelial migration is the key process in angiogenesis and VEGF_165_ plays crucial role in cell migration. To determine whether AuNPs could inhibit VEGF_165_-induced endothelial cell migration, wound-healing and Transwell monolayer permeability assays were employed to explore the changes in migration following treatment with VEGF_165_ that had been preincubated with or without AuNPs.

**Figure 2:**
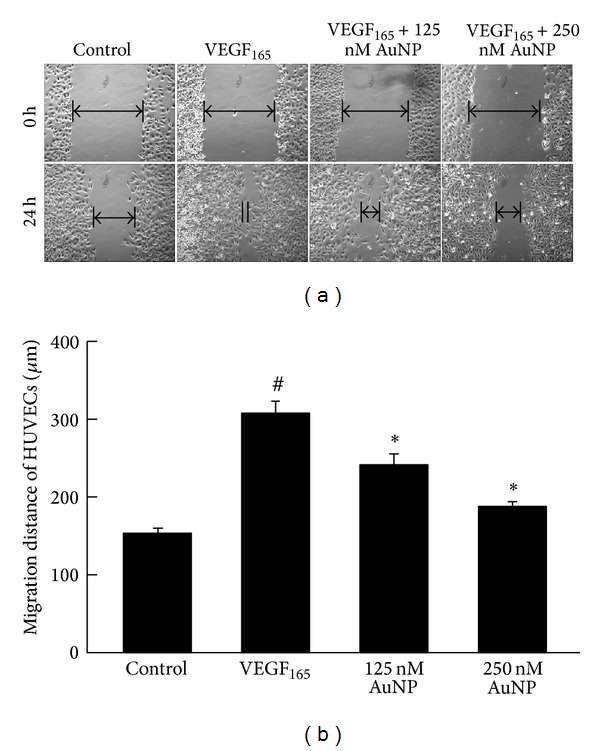
The effects of AuNPs on VEGF_165_-induced HUVEC migration. (a) Confluent HUVEC monolayers were wounded with pipette tips (upper panel) and treated with VEGF_165_ (20 ng/mL) in the presence or absence of AuNPs (125 or 250 nM). After 24 h incubation (lower panel), the wound closures were imaged to analyze the abilities of the cells to migrate into and fill the wounded areas. HUVECs incubated in 0.5% serum were used as controls. HUVECs treated with VEGF_165_ migrated efficiently from the wound margin relative to the controls. AuNPs significantly inhibited VEGF_165_-induced cell migration. (b) The average migration of cells treated with 0.5% serum (control) and VEGF_165_ (20 ng/mL) in the presence or absence of AuNPs (125 or 250 nM). Data are presented as the means ± SEM based on three independent experiments. ^#^ *P* < 0.05 compared with the control group; *P < 0.05 compared with the VEGF_165_ group.

As shown in Figure 2(a), the endothelial cell migration distance was 307.7 ± 18.9 *μ*m after VEGF_165_ treatment for 24 h; this represented a significant increase relative to that of the control group (152.3 ± 6.1 *μ*m, *P* < 0.01; Figure 2(b)). However, when HUVECs were treated with VEGF_165_ that had been preincubated with 125 nM or 250 nM AuNPs, the cell migration distance was significantly reduced to 241.6 ± 11.5 *μ*m or 188.3 ± 4.0 *μ*m, respectively (*P* < 0.01). These data show that AuNPs reduced endothelial cell migration distance in concentration-dependent manner.

**Figure 3:**
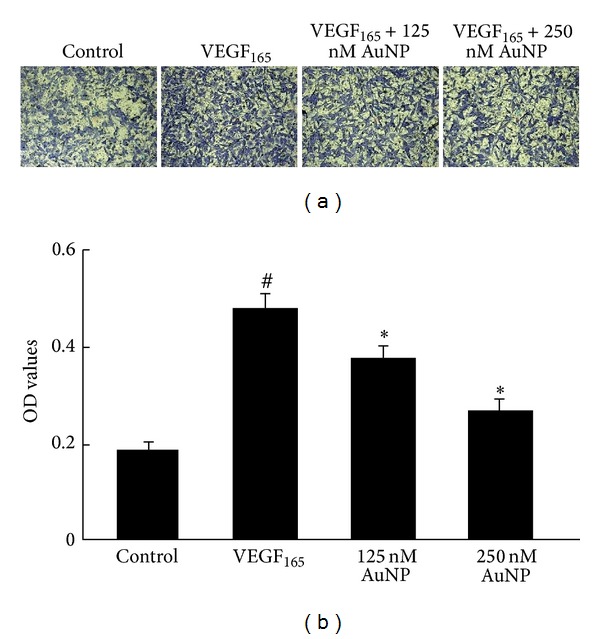
The effects of AuNPs on HUVEC migration in a Transwell assay. (a) Sample Transwell assay images showing the inhibitory effects of AuNPs on VEGF_165_-induced HUVEC migration after staining the transmigrated cells with crystal violet (magnification, 10x). VEGF_165_-treated (20 ng/mL) cells transmigrated through the Matrigel-coated Boyden chamber within 12 h, compared with controls, whereas pretreatment with AuNPs (125 or 250 nM) significantly reduced cell migration. (b) Quantification of the Transwell assay results: crystal violet OD values represent the amounts of transmigrated cells. Data are presented as the means ± SEM based on three independent experiments. ^#^ *P* < 0.05 compared with the control group; *P < 0.05 compared with the VEGF_165_ group.

We further used a Transwell system to examine the effects of AuNPs on VEGF_165_-induced endothelial cell migration. Compared with the nonstimulated control, a large number of HUVECs migrated to the lower side of the filter after stimulation with VEGF_165_ for 12 h, whereas AuNPs significantly prevented this migration in a concentration-dependent manner (Figure 3(a)). As shown in Figure 3(b), after VEGF_165_ treatment, the crystal violet OD values (representative of the amounts of migrated cells) increased from 0.19 ± 0.01 to 0.48 ± 0.03; however, treatment with VEGF_165_ after preincubation with 125 nM or 250 nM AuNPs reduced the crystal violet OD values to 0.38 ± 0.02 or 0.27 ± 0.02, respectively (*P* < 0.05; Figure 3(b)). These results confirm that AuNPs reduce crystal violet OD values in concentration-dependent manner. The above results suggest that AuNPs repress the VEGF_165_-induced cell migration.

### 3.3. Effects of AuNPs on VEGF_165_-Induced HUVEC Tube Formation

**Figure 4:**
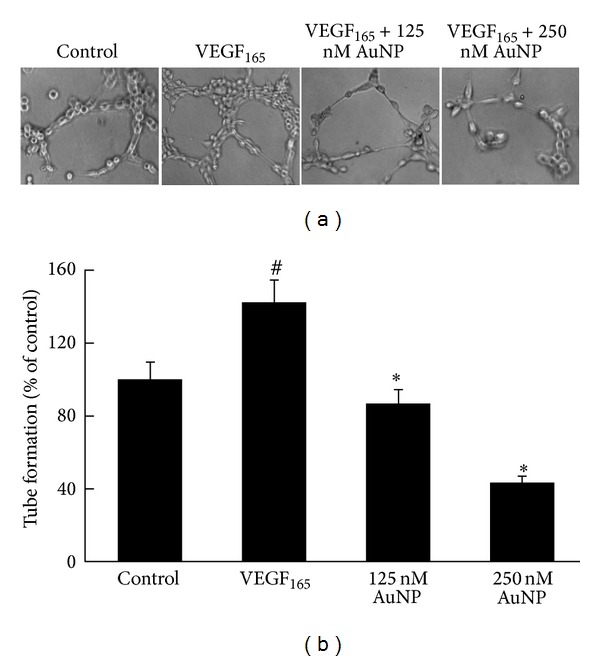
Effects of the AuNPs on VEGF_165_-induced HUVEC tube formation. (a) Sample images showing the inhibitory effect of AuNPs on VEGF_165_-induced HUVEC tube formation (magnification, 10x). HUVECs (1 × 10^5^ cells) were inoculated on the Matrigel surface and treated with VEGF_165_ (20 ng/mL) in the presence or absence of AuNPs (125 or 250 nM). (b) Tube formation was quantified by counting the connected cells in randomly selected fields at 100x magnification. Data are presented as the means ± SEM based on three independent experiments. ^#^ *P* < 0.05 compared with the control group; *P < 0.05 compared with the VEGF_165_ group.

Another important step in the angiogenic process is tubule formation. We used a three-dimensional Matrigel assay to examine the potential effects of AuNPs on VEGF_165_-induced tube formation. When HUVECs were placed on the Matrigel, robust, elongated tube-like structures formed after incubation in the presence of VEGF_165_ (Figure 4(a)). The number of formed tubules was calculated using inverted-phase contrast microscopy, which directly revealed the ability of the HUVECs to form tubular structures. As expected, VEGF_165_ clearly promoted tube formation, whereas the AuNPs significantly inhibited VEGF_165_-dependent tube formation. As shown in Figure 4(b), the tube-formation rate after VEGF_165_ treatment was approximately 142 ± 12.1% (relative to the control); however, tube formation in the presence of 125 nM or 250 nM AuNPs was reduced to 87 ± 7.4% or 43 ± 4.3% of the control, respectively (*P* < 0.05; Figure 4(b)). These results demonstrated the potential of AuNPs to inhibit VEGF_165_-induced tube formation.

### 3.4. Effects of AuNPs on CAM Angiogenesis

**Figure 5:**
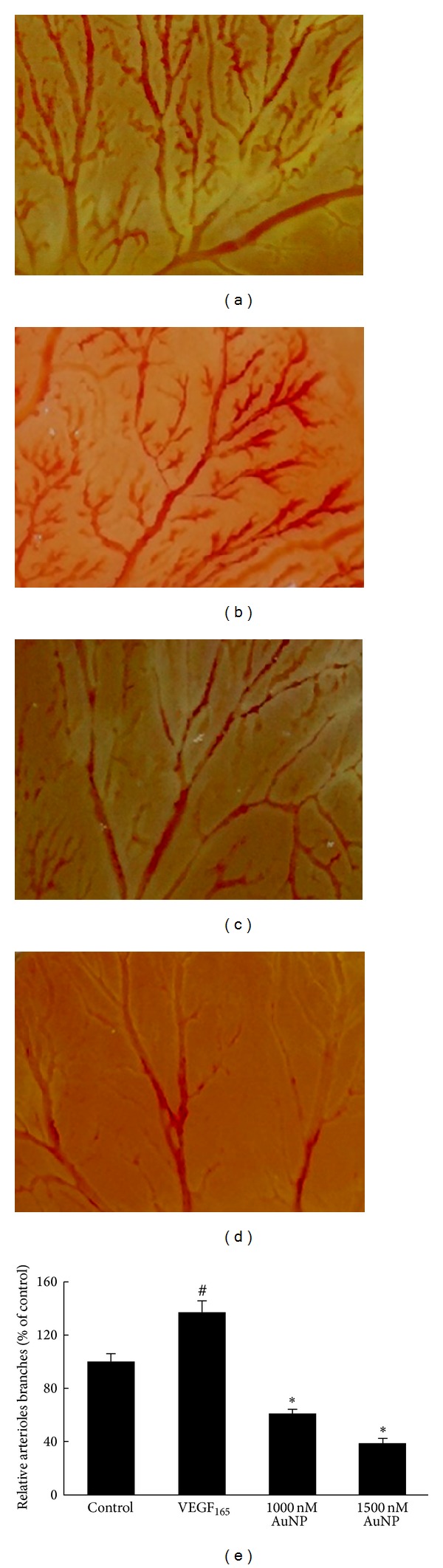
The effects of AuNPs on angiogenesis in the CAM. Fertilized eggs were treated with VEGF_165_ in the presence or absence of AuNPs for 48 h and subsequently harvested and photographed. (a) Control; (b) VEGF_165_ (80 nM/egg); (c) VEGF_165_ + AuNPs (1000 nM/egg); (d) VEGF_165_ + AuNPs (1500 nM/egg); (e) angiogenesis was quantified by counting the number of arteriole branches in each photograph. Data are presented as the means ± SEM based on three independent experiments. ^#^ *P* < 0.05 compared with the control group; *P < 0.05 compared with the VEGF_165_ group.

Because AuNPs inhibited VEGF_165_-induced tube formation in vitro, we next sought to examine whether they could also inhibit angiogenesis in vivo. The chicken chorioallantoic membrane (CAM) assay is a powerful in vivo angiogenesis model that we used to examine the effect of AuNPs on the formation of new capillaries. As shown in Figures 5(a)–5(d), angiogenesis was clearly observed in the fertilized eggs after a 48 h treatment and VEGF_165_ significantly promoted the formation of branched blood vessels in comparison to the untreated CAMs. A 48 h AuNP treatment dramatically decreased the numbers of branched vessels, particularly smaller vessels. The quantitative data are summarized in Figure 5(e). The branched blood vessel formation rate in the VEGF_165_-treated group was approximately 137 ± 8.7% (relative to the control group); however, after preincubation with 1000 nM or 1500 nM AuNPs, the rate decreased to 61 ± 3.1% or 39 ± 2.8%, respectively (*P* < 0.05). These results demonstrated that angiogenic development of the CAM arterial endpoint was stimulated by VEGF_165_ and inhibited by AuNPs.

### 3.5. Changes in the Cellular Ultrastructure as Detected by AFM

**Figure 6:**
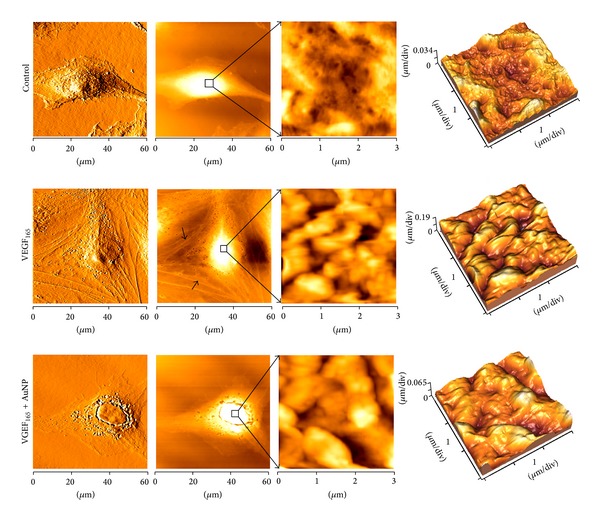
Changes in the cellular ultrastructure as detected by AFM. HUVECs were seeded onto slides and treated with VEGF_165_ (20 ng/mL) in the presence or absence of AuNPs (250 nM) for 24 h. The morphological changes of the HUVECs were assessed using AFM as described previously.

Atomic force microscopy is a useful tool for the collection of cellular surface information because it can detect the cellular topography, ultrastructure, and morphological changes [17, 18]. In this study, AFM was used to observe a variety of changes in the HUVEC surface morphology and ultrastructure in the different treatment groups. More than 5 cells per group were scanned and measured by AFM, although only representative images are presented. As shown in Figure 6, the nonstimulated HUVECs had fusiform morphology with smooth surfaces and few particles. VEGF_165_-treated HUVECs adhered closely to the substrate; these cells were more active and had a pleomorphic appearance with obvious pseudopodia (indicated by the black arrows in Figure 6), larger particles, and rough surfaces. In contrast, the HUVECs that had been incubated with AuNP-treated VEGF_165_ had a similar appearance to those of the control group, with fewer pseudopods and particles and a smoother cell surface. Furthermore, VEGF_165_-treated HUVECs exhibited considerable cellular plasmodesmata under AFM, whereas those incubated with AuNP-treated VEGF_165_ exhibited comparatively and significantly thinner plasmodesmata. This finding suggested that AuNPs could significantly block the VEGF_165_-induced cellular extension or migration.

Additionally, the particle size distributions and surface roughness in each cellular ultrastructure image (3 *μ*m × 3 *μ*m) were analyzed using software (Image Processing 2.1, IP 2.1). The statistical analyses of the particle sizes and the peak to valley roughness (Rp-v), the root-mean-square roughness (Rq), and the average roughness (Ra) of the cell surface are shown in Table 1. The results indicated that VEGF_165_ induced larger particle formation (*P* < 0.01) and increased surface roughness (*P* < 0.05) in the HUVECs, whereas AuNPs suppressed these changes and led to smaller particle formation (*P* < 0.01) and smoother surfaces (*P* < 0.05).

AuNPs inhibit VEGF_165_-induced HUVEC migration mediated by Akt pathway.

**Figure 7:**
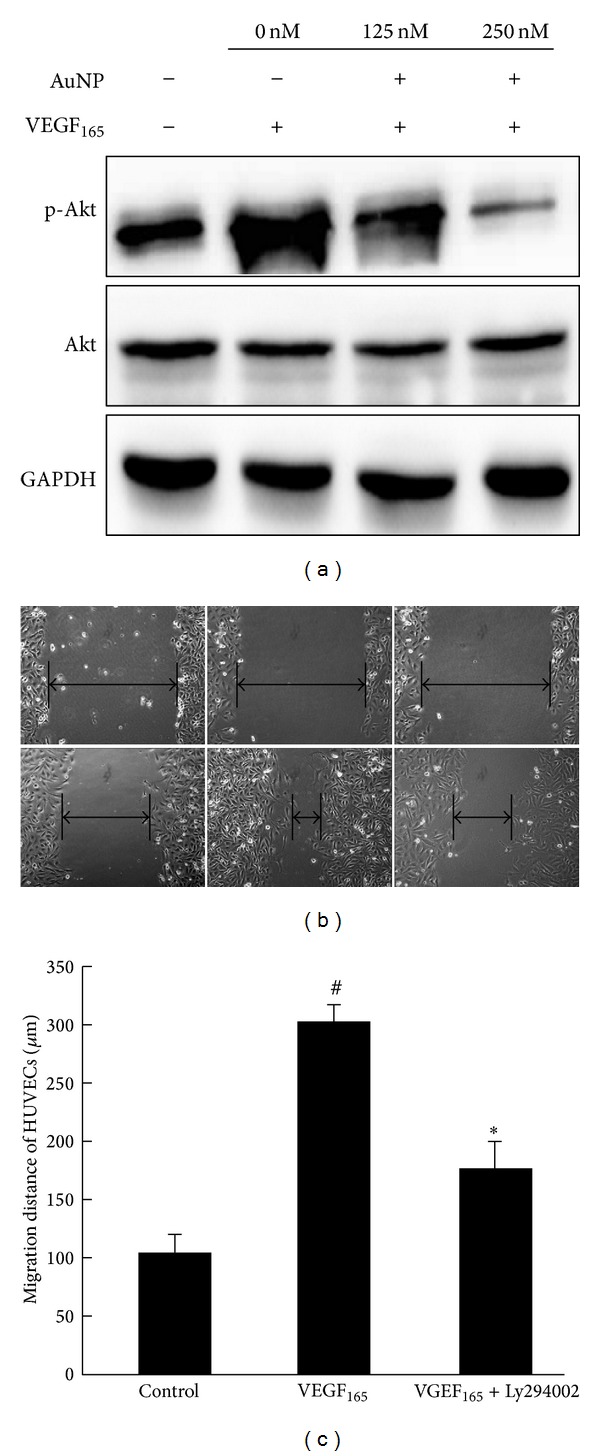
AuNPs inhibit VEGF_165_-induced HUVEC migration mediated by Akt pathway. (a) Serum-starved HUVECs were treated with VEGF_165_ (20 ng/mL) in the presence or absence of gold nanoparticles. The effects of AuNPs on phosphorylated Akt and pan Akt were determined using Western blot analysis. (b) Confluent HUVEC monolayers were wounded with pipette tips (upper panel) and treated with VEGF_165_ (20 ng/mL) in the Ly294002 (10 *μ*M). After 24 h incubation (lower panel), the wound closures were imaged to analyze the abilities of the cells to migrate into and fill the wounded areas. HUVECs incubated in 0.5% serum were used as controls. (c) The average migration of cells treated with 0.5% serum (control) and VEGF_165_ (20 ng/mL) in the Ly294002 (10 *μ*M). Data are presented as the means ± SEM based on three independent experiments. ^#^ *P* < 0.05 compared with the control group; *P < 0.05 compared with the VEGF_165 _ group.

VEGFR2 plays a major role in VEGF-dependent angiogenesis. Our previous study [13] showed that AuNPs significantly inhibited phospho-VEGFR2 induced by VEGF_165_. To investigate the mechanism associated with the AuNP-mediated inhibitory effects on VEGF_165_-induced migration and tube formation, we used Western blotting to examine the activation of Akt, which is an important downstream component of the VEGFR-2/PI3K signaling pathway in HUVECs. As shown in Figure 7, Akt phosphorylation was significantly increased in the HUVECs after VEGF_165_ treatment but significantly reduced in a dose-dependent manner after treatment with VEGF_165_ that had been preincubated with AuNPs. Next, a specific inhibitor of PI3K/Akt pathway, LY294002, was used to inhibit Akt phosphorylation in wound-healing assay to test whether inhibition of Akt phosphorylation has effect on VEGF_165_-induced migration. As shown in Figure 7(c), after VEGF_165_ treatment with 24 h, the endothelial cell migration distance significantly increased to 302.8 ± 17.2 *μ*m compared with that of the control group (115.3 ± 16.5 *μ*m, *P* < 0.05). However, when HUVECs were preincubated with 10 *μ*M Ly294002, the cell migration distance was reduced to 178.9 ± 19.8 *μ*m (*P* < 0.05). The results suggested that the AuNPs significantly inhibited VEGF_165_-induced Akt phosphorylation in the HUVECs, thus resulting in the inhibition of VEGF_165_-induced HUVEC migration.

## 4. Discussion

Angiogenesis, the growth of new capillaries from preexisting vessels, occurs as a result of dynamic endothelial cell functions such as migration and tube formation that are essential to the organized formation of vessel sprouts [3, 19]. We, therefore, evaluated these two critical angiogenic processes in this study. First, we examined whether AuNPs had any effect on VEGF_165_-induced HUVEC migration; the results of a wound-healing assay indicated a significant area of the uncovered wound after AuNPs treatment relative to that of the controls, and a Transwell monolayer permeability assay showed that the number of HUVECs that migrated to the lower side of the filter decreased after AuNPs treatment. Second, we observed the effects of AuNPs on VEGF_165_-induced HUVEC tube formation; the results of a Matrigel assay demonstrated that AuNPs inhibited or delayed the formation of HUVEC tube-like structures, and the results of a chick chorioallantoic membrane assay also revealed that AuNPs suppressed new capillary formation. The present report, therefore, demonstrates the inhibitory effects of AuNPs on VEGF_165_-induced HUVEC migration and tubule formation.

During neovascularization, vascular endothelial cell proliferation and migration occur initially, followed by organization into a network of tube-like structures and the formation of new capillaries [20]. During these complex processes, VEGF is considered the most important proangiogenic factor; in particular, VEGF_165_ and VEGF_121_ play major regulatory roles in the functions of vascular endothelial cells [4, 21]. A previous study reported that AuNPs could bind to VEGF_165_ through its heparin-binding domain but could not bind to VEGF_121_, which does not contain a heparin-binding domain [12, 13, 22]. Consequently, VEGF_165_ was used to stimulate HUVECs in all of the present experiments.

Reportedly, VEGFR-2 (Flk-1) is the main receptor through which VEGF mediates its biological effects in endothelial cells [23, 24]. VEGF_165_ can act through VEGFR-2 to mediate a signaling cascade that regulates all of the key steps of the angiogenic process, including endothelial cell division, proliferation, migration, and tube formation [25]. Several studies have suggested that the processes involved in the formation of new blood vessels, including proliferation, migration, and tube formation, could be suppressed by blocking VEGF signaling [15, 19, 25, 26]. The PI3K/Akt pathway is considered very important among the VEGF/VEGFR-2 downstream signaling pathways [27]. This pathway can regulate endothelial cell migration via the VEGFR-2/PI3K/Akt/eNOS axis [5, 6]. Akt (protein kinase B), a serine/threonine-specific protein kinase, regulates endothelial nitric oxide synthase activation to stimulate vasodilation, vascular remodeling, and angiogenesis [9, 20, 28]. Akt plays a critical role in endothelial cell migration and can be activated through the specific binding of VEGF_165_ to VEGFR-2, which then triggers the downstream VEGFR-2/PI3K/Akt signaling pathway. In this study, we used Western blotting to examine Akt phosphorylation and found that AuNPs significantly reduced VEGF_165_-induced Akt phosphorylation. In summary, it seems possible that AuNPs could inhibit VEGF_165_-induced HUVEC migration and tube formation by blocking the Akt signaling pathway subsequent to high-affinity binding to the heparin-binding domain of VEGF_165_, thus preventing the VEGF_165_-VEGFR-2 interaction. As a result, VEGF_165_ was inactivated by AuNPs, and the VEGF-mediated HUVEC stimulation on HUVECs was weakened, thus inhibiting the migratory and tube forming abilities.

Changes in cell morphology and structure are closely related to a cell's functional status; for example, endothelial cell morphological changes can be observed during proliferation, migration, or even tube formation [17]. What changes in cell morphology and structure can be observed in activated HUVECs? Therefore, it is very important to study the morphology and ultrastructure of HUVECs to investigate migration or tube formation. We observed the cell membrane surface ultrastructures using atomic force microscopy (AFM), which allowed the cellular changes after VEGF_165_ treatment in the presence or absence of AuNPs to be observed visually. The AFM images revealed increased HUVEC activity in response to VEGF_165_ treatment and cells with pleomorphic appearance, obvious pseudopodia, larger particles, and increased cell membrane surface roughness; in contrast, the HUVECs appeared to be less active after AuNP treatment. These results indicated that AuNPs could significantly block VEGF_165_-induced cellular proliferation or migration. AFM is a powerful method that we can with which image cell surface ultrastructures under physiological conditions [29]. As a nondestructive surface imaging tool, AFM can obtain nanometric-scale images of the cell surface and can provide very important morphological and cell membrane details [18, 30]. Changes in cell morphology and pseudopod formation indicate the level of cell activity, and the cell surface particles indicate metabolic activity and functional protein secretion [31]. Endothelial cell migration and morphological changes are essential for angiogenesis [6]. Therefore, the changes in cellular morphology and surface ultrastructure that were detected in this study might intuitively reflect the migratory and tube forming abilities of the HUVECs and could thus provide new insights into the angiogenic process in these cells.

In summary, our study focused on the inhibitory effects of AuNPs on HUVEC migration, CAM, and tube formation, the key angiogenic characteristics of endothelial cells. Moreover, we used AFM to observe changes in cellular micromorphology. The early stages of neovascularization can be divided into three well-known steps: endothelial cell proliferation, migration, and tube formation; these are regulated by VEGF and are also the main targets of antiangiogenesis [15, 25]. We previously reported that AuNPs could inhibit VEGF_165_-induced HUVEC proliferation by binding to the sulfur and amine groups present in the heparin-binding domains of VEGF_165_ [13]. In this study, we observed that AuNPs could also inhibit two other angiogenic steps.

## 5. Conclusions

In conclusion, our study demonstrated that AuNPs could significantly inhibit VEGF_165_-induced HUVEC migration and tube formation both in vitro and in vivo and provided the first nanoscale images of the AuNP-mediated inhibition of HUVEC cell migration and tube formation. We speculate that AuNPs might inhibit these processes in Akt signaling pathway-mediated processes because the particles bind to VEGF_165_ and thus indirectly reduce VEGFR-2 activation. These findings have provided new insights into the antiangiogenic effects of AuNPs.

## Figures and Tables

**Table 1:** Statistical analyses of the particle sizes and the cell surface Rp-v, Rq, and Ra (mean ± SD; nm).

Group	Particle size	Rp-v	Rq	Ra
Control group	57.40 ± 4.61	71.73 ± 5.77	9.94 ± 2.87	7.97 ± 2.70
VEGF_165_ group	278.67 ± 17.01	348.43 ± 20.83	53.10 ± 7.90	42.76 ± 7.62
VEGF_165_ + AuNP group	94.97 ± 9.24	118.57 ± 11.37	17.79 ± 3.43	13.70 ± 3.07

*P* values	0.000*, 0.000^Δ^	0.000*, 0.000^Δ^	0.017*, 0.023^Δ^	0.027*, 0.038^Δ^

*P: control group versus VEGF_165_ group; ^Δ^ *P*: VEGF_165_ group versus VEGF_165_ + AuNP group.
